# Supplementary material for: The genomic and ecological context of hybridization affects the probability that symmetrical incompatibilities drive hybrid speciation
Source: Ecol Evol. 2018 Feb 14;8(5):2926–37. doi: 10.1002/ece3.3872 (PMC5838063; doi:10.1002/ece3.3872)
Supplement: Supplementary file 1 [file ECE3-8-2926-s001.docx]

**Supplementary Figures**

Figure S1. Selection at linked sites and the evolution of symmetrical incompatibilities. Companion figure to Figure 4 of the main text. Results are shown for populations simulated with inter-locus recombination rates of 0.5 (i.e. no linkage; left column of panels) or 0.1 (moderate linkage; right column of panels), *m* = 0.001, and with different linear arrangements of loci along the chromosome (i.e. genetic architectures; panel rows).

Figure S2. The proportion of hybrid populations regressing to P1 ancestry across all epistatic loci as a function of *s*_adaptive ­_(increasing along the x-axis) and *s*_epistatic_ (different colored lines) with directional selection acting on adaptive loci. Panels are arranged such that migration rates increase from left to right and genetic architecture varies down the panel columns. Results are shown for *r* = 0.5 between adjacent loci (i.e. no linkage). See Figures S3 and S4 for results with moderate (*r* = 0.2) and stronger linkage (*r* = 0.1), respectively.

Figure S3. The proportion of hybrid populations regressing to P1 ancestry across all epistatic loci as a function of *s*_adaptive ­_(increasing along the x-axis) and *s*_epistatic_ (different colored lines) with directional selection acting on adaptive loci. Panels are arranged such that migration rates increase from left to right and genetic architecture varies down the panel columns. Results are shown for *r* = 0.2.

Figure S4. The proportion of hybrid populations regressing to P1 ancestry across all epistatic loci as a function of *s*_adaptive ­_(increasing along the x-axis) and *s*_epistatic_ (different colored lines) with directional selection acting on adaptive loci. Panels are arranged such that migration rates increase from left to right and genetic architecture varies down the panel columns. Results are shown for *r* = 0.1.

Figure S5. A modular genetic architecture (bottom row of panels) can facilitate the evolution of symmetrical incompatibilities when there is strong direction selection favoring parental alleles derived from one parent at linked adaptive loci (increasing values on the x-axis). Results are shown for simulations initiated with a forced bout of hybridization (i.e. individuals in the ‘hybrid deme’ were all F1s) and moderate linkage between loci (*r* = 0.2).

Figure S6. A modular genetic architecture (bottom row of panels) can facilitate the evolution of symmetrical incompatibilities when there is strong disruptive selection favoring parental haplotypes over hybrid and admixed haplotypes at linked adaptive loci (increasing values on the x-axis). Results are shown for simulations initiated with a forced bout of hybridization (i.e. individuals in the ‘hybrid deme’ were all F1s) and moderate linkage between loci (*r* = 0.2).

Figure S7. Linkage between epistatic and adaptive loci (i.e. dispersed and interspersed genetic architectures; top two panel rows) can facilitate the evolution of symmetrical incompatibilities when there is selection-for-admixture at adaptive loci (increasing values on the x-axis). Results are shown for simulations initiated with a forced bout of hybridization (i.e. individuals in the ‘hybrid deme’ were all F1s) and moderate linkage between adjacent loci (*r* = 0.2).
